# David Rall and the National Toxicology Program

**DOI:** 10.1289/ehp.113-a152b

**Published:** 2005-03

**Authors:** James Huff

**Affiliations:** National Institute of Environmental, Health Sciences, Research Triangle Park, North Carolina, E-mail: huff1@niehs.nih.gov

Concerning the 25 year history and milestones of the National Toxicology Program (NTP), McGovern (2004) failed to acknowledge the huge conceptual and leadership contributions provided by David P. Rall (1926–1999) in the creation, development, and continuing achievements of the NTP (Huff 2000). Of course, Rall was the individual most responsible for conceiving, nurturing, and establishing the National Institute of Environmental Health Sciences (NIEHS) as a world-recognized leader in environmental health sciences. The NTP, a natural and independent partner of the NIEHS, was the innovative idea of Rall, who with a few other like-minded collegial giants in the fields of public and occupational health, recognized the need to better coordinate the disparate and often redundant toxicology and health hazard identification activities in the Department of Health and Human Services.

Among others, Congressman David Obey (Wisconsin) was convinced by Rall and key colleagues that a coordinated national program was needed to better promote the health of the American people by protecting them from exposures to hazardous chemicals in the workplace, environment, and home. Obey was instrumental in getting Congress to endorse this need for a national program and, after Rall’s untimely death, encouraged Congress to name the main structure housing NIEHS/NTP in Research Triangle Park, North Carolina, as the David P. Rall Building.

Rall was supported in this innovative and monumental NTP-forming effort by several other distinguished pioneers in environmental health research and public health, including Eula Bingham, then director of the Occupational Safety and Health Administration; Joseph Califano, then Secretary of the Department of Health, Education, and Welfare (renamed the Department of Health and Human Services); Donald Fredrickson, then director of the National Institutes of Health; Donald Kennedy, then director of the Food and Drug Administration; Cesare Maltoni, then director of the Bologna Centre for the Prevention and Detection of Tumours and Oncological Research; Norton Nelson, then director of the Institute of Environmental Medicine (renamed the Nelson Institute of Environmental Medicine) New York University School of Medicine; Irving Selikoff, then director of the Mt. Sinai Medical Center in New York City (renamed Mount Sinai-Irving J. Selikoff Clinical Center for Occupational and Environmental Medicine); Lorenzo Tomatis, then chief of the Unit of Chemical Carcinogenesis, International Agency for Research on Cancer (later director of IARC); and Arthur Upton, then director of the National Cancer Institute.

Also, during that NTP-formative era, Rall helped establish the 1978 Public Law that initiated the innovative *Report on Carcinogens* (Huff 1998). Now in its eleventh edition, the RoC documents 246 chemicals, groups of chemicals, or mixtures known or anticipated to cause cancer in humans. Much earlier, in 1972, Rall originated the pivotally directed and most frequently referenced environmental journal, *Environmental Health Perspectives*. Of course, during his illustrious career as physician, assistant surgeon general, scientist, and staunch public health advocate, Rall made many more and varied contributions to basic sciences and public health (Hinson 2000; Huff 2000, 2002; Rall 2000).

David P. Rall, a dedicated physician and scientist, is among a relativity small group of exceptional public servants who have had a deep and lasting positive impact on human health by showing us how to understand and improve the environment in which we live, work, and play. To many of us who joined with him to make a safer environment, we clearly recognize the void he has left.
